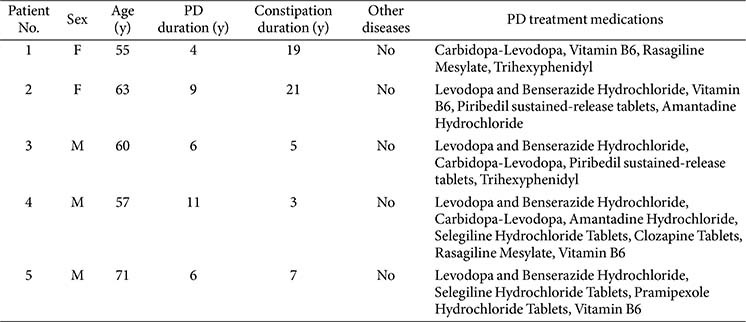# Pilot Clinical Trial of Fecal Microbiota Transplantation for Constipation in Parkinson's Disease

**DOI:** 10.4014/jmb.2509.09029

**Published:** 2025-12-29

**Authors:** Huilu Zhang, Cong Shen, Wei Lei, Jian Wang, Jun Liu, Zhibing Qiu

**Affiliations:** 1 Department of Digestive Diseases of Huashan Hospital, Fudan University, Shanghai 200040, P.R. China; 2 Department of Neurology and National Research Center for Aging and Medicine & National Center for Neurological Disorders, State Key Laboratory of Medical Neurobiology, Huashan Hospital, Fudan University, Shanghai, P.R. China; 3 Department of Nursing, Huashan Hospital, Fudan University, Shanghai, P.R. China

**Keywords:** Parkinson disease, constipation, fecal microbiota transplantation, metabolomics, metagenomics

## Abstract

The purpose of this study was to evaluate the safety and efficacy of fecal microbiota transplantation in patients with constipation due to parkinson’s disease. Gut dysbiosis has long been associated with parkinson’s and recent studies have shown that FMT can restore the normal flora of the gut. Therefore, this clinical trial aimed to test the therapeutic efficacy of FMT in 5 patients aged 55 to 71 diagnosed with PD who presented with constipation. The study was conducted as an open label, prospective trial and consisted of FMT performed every 3 days via nasojejunal tube placement followed by 8 weeks of patient follow-up to evaluate response to drug therapy and to assess neurological function using UPDRS-III OFF scores, and improvement in constipation assessed with Wexner scores. Samples taken before and after FMT were collected for shotgun metagenomic sequencing to analyze the composition of the microbial communities present in patients. Untargeted non-targeted metabolomic studies were performed to investigate the impact of FMT on metabolome changes due to FMT. The results indicate an improvement in constipation and neurological functioning following FMT, and significant alteration of the gut microbiota. Significant increases in *Bifidobacteria bifidus*, *Alistipes shahi*, *Anaerotruncus coli*, and uncharacterized *Flavonifractor* were found post-treatment compared to the baseline. Many of the other strains present prior to treatment, including *Acinetobacter* sp. and *Proteobacteria* sp., had significantly decreased after the FMT. The metabolomic studies found shifts in metabolic pathways involved with unsaturated fatty acid synthesis and amino acid metabolism due to FMT. FMT may be an effective treatment option for constipation and neurological symptoms associated with PD.

## Introduction

Parkinson's disease (PD) is a major public health and social problem because it affects so many people and causes so many disabilities[1]. This neurodegenerative disorder is typically characterized by motor dysfunctions, such as myotonia, static tremor, an abnormal gait and posture, and bradykinesia. These clinical manifestations are due to gradual loss of dopaminergic neurons within the substantia nigra pars compacta (SNpc) of basal ganglia and abnormal deposition of α-Synuclein (α-Syn) containing Lewy bodies [2] and they worsen overtime. On the other hand, non-motor symptoms usually precede motor symptoms for years, including constipation, pain, depression, and REM sleep behavior disorder (RBD) [3]. PD is known to involve multiple molecular and cellular processes during progressing, thus making it difficult for early intervention and efficient treatment. Current treatment strategies include medications on the central dopamine system, deep brain stimulation (DBS), gene therapy and stem-cell therapy [4].

Gastrointestinal manifestations, including flatulence, constipation, defecation disorders, and impaired gastrointestinal motility, are commonly observed in patients with PD. The incidence of constipation in PD, approximately 40%~50%, is most commonly occurred during the prodromal stage [5]. The gut-originated theory of PD is brought up by Braak *et al*. that α-Syn aggregates originate from the enteric nervous system (ENS) and route along vagal nerve to the hindbrain [6]. This have been supported by findings on post-mortem specimens that phosphorylated α-Syn can be spotted around vagal ganglia and sub-mucosal nerve endings at stomach, duodenum and colon [7]. A comparison study following patients from gastric vagotomy surgery also reveals that those who undergo total vagotomy are less susceptible to the development of PD [8]. Collectively, researches on gut-brain axis regulation of PD are gaining great interests and offer a unique perspective on the etiology of PD.

The role of gut microbiota has been linked to various neurological disorders, including PD [9, 10]. Studies concerning microbiota profiles with PD patients are controversial due to the heterogeneity in different clinical cohorts. Nevertheless, researchers have reached a consensus on a reduction of α- and β-diversity of bacterial abundance with PD patients. To be specific, certain strains like *Akkermansia* are reported to be increased in relative abundance, while the relative abundance of *Prevotella* and other strains are decreased [11]. The alterations in microbiota landscape would compromise the mucosal barrier and harm the balance of immune system. Some bacterial species can promote the accumulation of α-synuclein and correlate with increased intestinal permeability [12]. FMT has been successfully applied on the treatment of refractory inflammatory bowel disease, *Clostridium difficile* infection, refractory intestinal infection, and other digestive diseases through the reconstruction of commensal microbiota of recipients [13-15]. FMT has great potentials in treating PD and helping improve patient care. Indeed, random controlled trials studies on FMT treatment of PD sprout during recent years and confirm the feasibility and effectiveness of FMT therapy [16, 17].

In the present work, we designed a self-controlled study to investigate the improvements of motor and non-motor symptoms before and after FMT treatment. More importantly, we combined metagenomic and metabolomic data to explore the correlation between microbiota-derived metabolites and clinical outcomes. We hope to not only reassure the safety and efficacy of FMT treatment on PD patients, but also discover possible microbiota-centered targets for further mechanical studies.

## Material and Methods

### Donors

Eleven volunteers were initially interviewed, and two were selected as potential donors; however, one was later excluded due to infection. Following the physical examination, one donor developed an upper respiratory tract infection and deemed ineligible to donate. Thus, five patients received FMT all from the preparation of one female donor, aged 21 years old, 162 cm in height, 58 kg in weight, and 22.1 in body mass index (BMI). The donor was in perfect health during the preparation of the study and had no familial history of diabetes, tumor, or allergies.

### Study Design and Patient Enrollment

A total of five patients with PD and concomitant constipation were enrolled in the clinical trial after providing written informed consent. Detailed demographic and clinical characteristics of the participants are presented in Table 1.

Subject selection criteria: (1) Meet the diagnosis of PD with constipation; (2) Age range: 40-75 years old; (3) Using multiple treatment methods before treatment did not show significant improvement in constipation; (4) Colonoscopy examination showed no obvious organic diseases; (5) Understand and sign the informed consent form.

Diagnostic criteria for PD: The primary core criterion for diagnosis is to clarify Parkinson's syndrome, defined as the presence of bradykinesia and at least one of the two main symptoms of resting tremor or rigidity. The examination of all core features must be conducted according to the methods described in the MDS-UPDRS Unified Parkinson's Disease Assessment Scale.

Diagnostic criteria for constipation in PD patients: Reduced frequency of defecation, dry and hard stool, and/or difficulty in defecation: Reduced frequency of defecation refers to less than 3 bowel movements per week; Difficulty in defecation includes difficulty in defecation, difficulty in excretion, incomplete defecation, time-consuming defecation, and the need for manual assistance in defecation; constipation. The course of illness is at least 6 months. The symptoms were evaluated using the Wexner Constipation Rating Scale.

### Exclusion Criteria for Subjects:

(1) Accompanied by other acute infectious diseases, such as viral hepatitis, tuberculosis infection, etc; (2) Constipation symptoms persist for less than 6 months; (3) Moderate to severe chronic obstructive pulmonary disease; (4) Abnormal liver and kidney function; (5) Patients with severe hypertension and cerebrovascular accidents; (6) patients with congestive heart failure and unstable angina pectoris; (7) Accompanying with other serious diseases that may hinder their enrollment or affect their survival, such as tumor or AIDS. (8) Patients with anxiety, depression, mental or legal disabilities; Suspect or have a history of alcohol/drug abuse; (9) Women of childbearing age who are preparing to become pregnant during the study period; (10)There are contraindications for colonoscopy examination, such as intestinal perforation, intestinal obstruction, etc; (11) Other patients deemed unsuitable for inclusion by the researchers.

The GenFMTer (FMT Medical, China) automated purification system's instructions were followed to clean and prepare the fecal microbiota suspension [18]. Patients received transendoscopic enteral tubing (TET) surgery and FMT through the nasojejunal tube for three consecutive days. A total amount of 100 ml of fecal microbiota suspension was injected for each day. Patients were kept in observation for vital signs, abdominal symptoms and defecation behavior for 24 h post the surgery. Patients were assessed using the Wexner Constipation Score [19] and the Unified Parkinson’s Disease Rating Scale (UPDRS) [20] before FMT treatment, as well as at 4 and 8 weeks post-FMT. Serum and fecal samples were collected prior to FMT and at 2, 4, 6, and 8 weeks following the procedure. They were evaluated by the Unified Parkinson’s Disease Rating Scale (UPDRS) score^19^ and Wexner constipation score [20] prior to FMT treatment and at 4 and 8 weeks post-FMT. Fecal and serum samples were collected prior to FMT treatment and at 2, 4, 6, 8 weeks post-FMT.

This study received approval from the Ethics Committee of Huashan Hospital, Fudan University (Approval No. 2018-415), and all procedures were conducted in accordance with established ethical guidelines. The clinical trial, titled “Exploratory Clinical Study of Fecal Microbiota Transplantation for the Treatment of Parkinson’s Disease” was registered with the China Clinical Trials Registry (ChiCTR1900027055; registered on 30 October 2019, www.chictr.org.cn), where detailed study information is publicly accessible. All participants were informed of possible adverse effects, provided written informed consent, and voluntarily underwent FMT.

### Metagenomic Profiling of Fecal Microbiota

Bacterial DNA was extracted from each stool sample with QIAamp Fast DNA Mini Kit (Qaigen, Germany) in accordance with the manufacturer’s protocol. We then used the Illumina TruSeq DNA Sample Prep v2 Guide (Illumina, Inc., USA) to make DNA libraries for each sample with insert sizes of about 500 bp. We used an Illumina X-ten platform (USA) to do 150 bp paired-end sequencing on all of the samples. This allowed us to align the clean reads to the NCBI database (National Center for Biotechnology Information) using SOAPaligner 2.21 to identify known bacteria, fungi, viruses, and archaea. Taxonomic relative abundance profiles at multiple levels were generated using BLASTS (version 2.2.28+), and aligned reads were classified into Kingdom, Phylum, Class, Order, Family, Genus, and Species.

After that, the EggNOG (Evolutionary Genealogy of Genes) and KEGG (Kyoto Encyclopedia of Genes and Genomes) databases were used to add information to genes that weren't already in the database. It was thought that the assembled protein sequence had the same function as the protein sequence in the database when the two sequences were similar (score >60 and E value <1e-5). We calculated the relative abundance of all orthologous genes to find out how common each KO/NOG is.

### Metabolomic Analysis of Fecal and Serum Samples

The metabolomics data acquisition for the fecal and serum samples was performed with a UHPLC system (Nexera UHPLC LC-30A, Shimadzu, Japan) coupled with mass spectrometer (Triple TOF 5600+, AB SCIEX, USA). Chromatographic separation was performed using a UPLC HSS T3 column (2.1 × 100 mm, 1.8 μm); positive and negative ion modes were represented as POS and NEG. Raw data was converted by AbfConverter software (version 4.0.0) and subsequently processed in MS-DIAL software for peak extraction, alignment, and filtering. Metabolites were identified by mass charge ratio (*m/z*) and retention time (RT). Following standardization, data matrix was subjected to multivariate statistical analysis using SIMCA-P software (version 14.0, Umetrics AB, Sweden). The search of differentially enriched metabolites was based on the VIP value of the OPLS-DA model (threshold >1) and *P*-value (*P* < 0.05). METLIN, the online metabolites database, was utilized for the identification of unknown metabolites [21]. We have uploaded the metagenomic data, the link is as follows: https://www.ncbi.nlm.nih.gov/sra/PRJNA1284285.

### Statistical Analysis

Statistical analyses between groups were performed using GraphPad Prism software (Prism v8.2.1, GraphPad Software, Inc.). Student’s *t*-test of paired two-tailed design was used to analyse data. Nonparametric Wilcoxon testing were used for small sample comparisons. *P* < 0.05 was considered significant and marked with "+". *P* < 0.01 was marked with "*". The correlative analyses of microbiota species and metabolites were carried out by R software (version 3.5.1). For the metabolomic analysis, VIP values from the OPLS-DA model (threshold > 1) were combined with *t*-test p-values (*P* < 0.05).

To quantitatively analyze differential metabolites, both the local mass spectrometry database and the online METLIN database were searched by matching mass-to-charge (m/z) ratios or exact molecular masses. Statistical analysis was performed in Prism v8.2.1 (GraphPad Software, Inc.) using a paired, two-tailed Student’s *t*-test to compare groups, with *P* < 0.05 considered statistically significant. Correlation between differential species and metabolites were assessed using species-level data in R (v3.5). When *P* < 0.05, significance was denoted with “+”, and when *P* < 0.01, with “*”. In the correlation plots, blue and red indicate negative and positive correlations, respectively, with deeper colors representing stronger relationship.

## Results

### FMT Ameliorates Neurological Symptoms and Constipation in PD

**Fig. 1:**
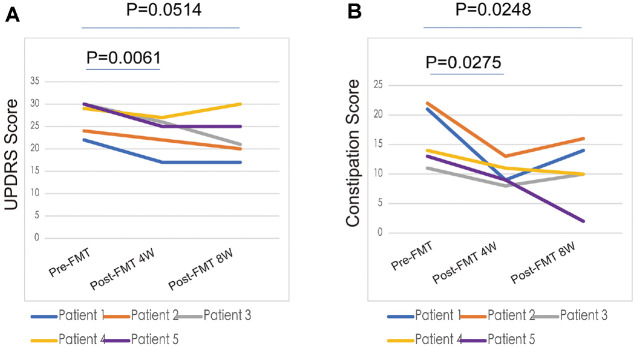
FMT reshapes the gut microbiota landscape of Parkinson's patients. (**A**) Changes in observed number of species and species abundance in the feces of patients before and after FMT were detected by shotgun metagenomic sequencing. (**B-C**) Changes in bacterial genus abundance at different time points before and after fecal bacteria transplantation. (**D-E**) Changes in bacterial species abundance at different time points before and after fecal bacteria transplantation. (**F**) Time-dependent changes in the abundance of different strains during FMT processes. Data are shown as mean ± SEM, *P < 0.05, **P < 0.01.

Five constipated PD patients signed informed consent forms and were enrolled in the FMT research trial. The UPDRS-III score was applied to measure how much better neurological symptoms got prior to and following FMT. All patients exhibited varying degrees of deterioration in their UPDRS-III OFF score during the follow-up period subsequent to FMT (Fig. 1A). Fig. 1A shows that the score showed a statistically significant difference in the fourth week, but by the eighth week, the difference was only getting smaller. Cases 1 and 5, on the other hand, showed a 5-point drop in their UPDRS-III OFF score 4 weeks after FMT (post-FMT 4W). This improvement lasted for another four weeks (post-FMT 8W). Eight weeks after FMT, we noticed that the individual in case 1 demonstrated a greater finger-tapping test and when they moved their left hand. Case 3's score, on the other hand, steadily went down from 30 to 21 over the course of the observation. The decrease in rigidity is the most obvious of the five UPDRS sub-scores, subsequently accompanied by alterations in movement speed and range. Even though case 4 showed no reduction in the UPDRS-III OFF score at follow-up, an interesting finding was observed. At the one month follow-up, the patient felt so well that he cut back on taking his anti-parkinsonian medications (Madopar and Sinemet from q.i.d. to t.i.d.) without talking to his doctor first. The changes in medication dosage affected the results noted at the post-FMT 8W; however, this self-statement illustrated the impact of FMT from an alternative perspective. According to the patient’s daughter (case 4), his daily functioning and memory had improved since undergoing FMT. The case 1 patient also said that her right shoulder pain and stiffness had gotten better.

Improvement in constipation were evaluated using the Wexner score before and FMT. Four weeks after FMT, all five patients saw a big improvement in their constipation symptoms (Fig. 1B). Four of these patients had constipation problems again and again, but the fifth patient said that things got better for a long time eight weeks later. Two patients had stomach pain after their FMT treatment, but they were able to fix it on their own within an hour without needing any more help. Diarrhea occurred more frequently after transplantation in one instance but rapidly resolved. Fever and hematochezia, which are other bad effects, did not happen.

All of these initial results indicated that constipation and neurological symptoms diminished during the first four weeks of FMT treatment. Individual variations led to disparities in the durability of enhancements.

### FMT Induces Significant Remodelling of the Gut Mirobiota in PD

Shotgun metagenomic sequencing of feces samples from five patients has been conducted to ascertain the difference as well as determine the change in their micro-biome after receiving fecal-microbiota transplantation (FMT). There is no change either in the number of bacteria per gram or how each patient’s population of bacteria related to another before and after transplanting fecal matter. There was a statistically significant increase in general species diversity within the second through fourth patients at comparison of their Pre-FMT versus FMT-Start, however subsequent comparisons of their popularity showed a decline in general diversity after that time.

**Fig. 2:**
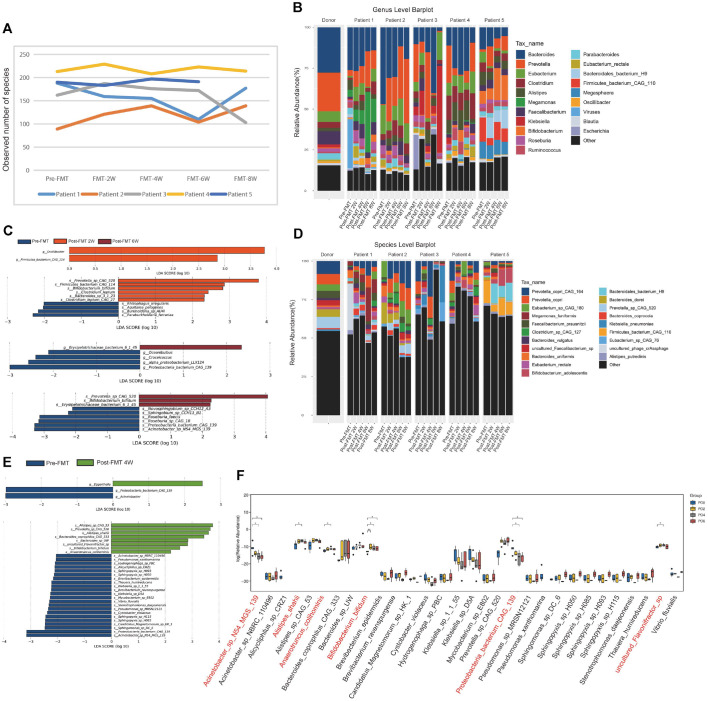
FMT impacts amino acid and fatty acid metabolism in Parkinson's patients. (**A**) Functional genomics analysis of gene structure changes in microbiota after FMT. (**B**) Heatmap showing significantly enriched metabolites in fecal samples 4 weeks after FMT. (**C**) Pathway enrichment that identified phenylalanine, tyrosine and tryptophan synthesis to be the most significantly enriched module in the fecal metabolites profile. (**D**) Heatmap showing significantly enriched metabolites in serum samples 4 weeks after FMT. (**E**) Pathway enrichment that identified biosynthesis of unsaturated fatty acids to be the most significantly enriched module in the serum metabolites profile.

The composition of the microbiome at the genus level was altered following fecal bacteria transplantation (FMT), with increases in the relative abundance of Firmicutes and *Oscillibacter bacterim* as of 2 weeks; Eggerthella as of 4 weeks; and *Erysipelotrichaceae bacterium* as of 6 weeks. Erysipelotrichaceae decreased by relative abundance from baseline levels in the 4-week and 6-week periods to those of the 2-week period, at which time there was no detectable Erysipelotrichaceae relative abundance. Analysis of the FMT-associated differences at the species level showed a significant amount of changes in relation to the fecal bacteria between the 2-week, 4-week, 6-week and 8-week periods following FMT (Fig. 2E). The greatest difference between the FMT and non-FMT periods was between the 2-week and 4-week periods (Fig. 2D) by LEfSe analysis (see Supplemental Data). The corresponding times for changes in the relative abundance of individual strains identified included increased numbers of uncultured *Flavonifractor* sp., *Proteobacteria bacterium* sp., *Anaerotruncus colihominis*, *Acinectobacter* sp., and *Bifidobacterium bifidum* following transplantation relative to baseline levels (Fig. 2F).

### FMT Alters Fatty Acid and Amino Acid Metabolism in PD

**Fig. 3:**
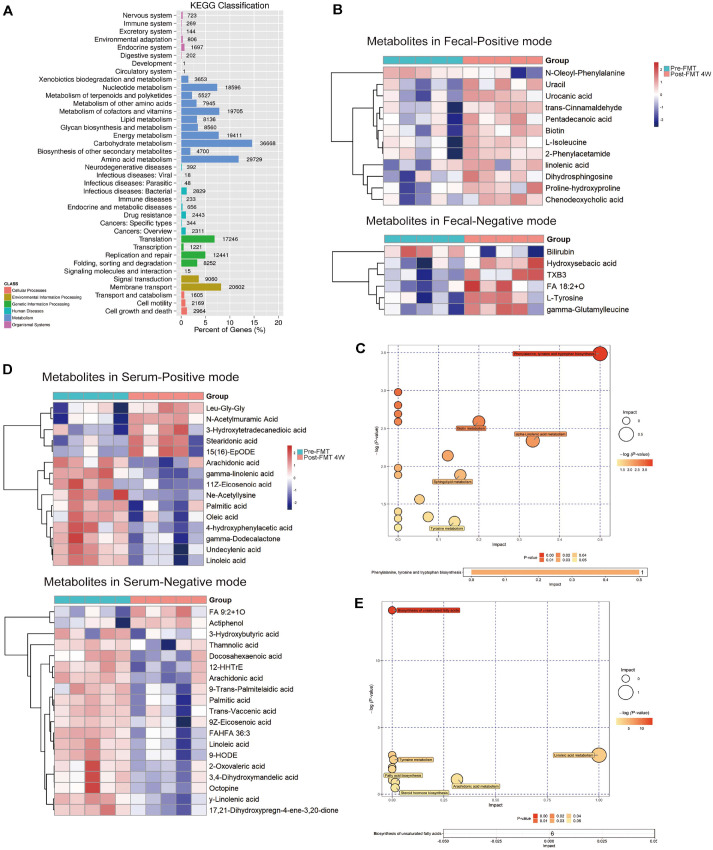
FMT impacts amino acid and fatty acid metabolism in Parkinson's patients. (**A**) Functional genomics analysis of gene structure changes in microbiota after FMT. (**B**) Heatmap showing significantly enriched metabolites in fecal samples 4 weeks after FMT. (**C**) Pathway enrichment that identified phenylalanine, tyrosine and tryptophan synthesis to be the most significantly enriched module in the fecal metabolites profile. (**D**) Heatmap showing significantly enriched metabolites in serum samples 4 weeks after FMT. (**E**) Pathway enrichment that identified biosynthesis of unsaturated fatty acids to be the most significantly enriched module in the serum metabolites profile.

Using ANOVA, we found gene modules that were very different between samples from donors, before FMT, and after FMT. Interestingly, pre-FMT samples were missing many modules that are important for the fundamental metabolism and production of carbohydrates, amino acids, and nucleotides (Fig. 3A). This was not the case with donor and post-FMT samples. We performed untargeted metabolomic analysis on serum and fecal samples from patients before and after FMT to elucidate metabolites and metabolic pathways implicated in the FMT process. Fecal metabolite analyses indicated that FMT significantly influenced the metabolism of phenylalanine, tyrosine, and tryptophan (Figs. 3B-3C and S3). This caused the levels of n-oleoyl-phenylalanine to go down and the levels of metabolites like L-isoleucine, L-tyrosine, indoleacetic acid (IAA), proline-hydroxyproline, γ-glutamylleucine, and 2-phenylacetamide to go up. It was also noted that FMT enhanced the fatty acid production in the gut lumen. These fatty acids include TXB3, chenodeoxycholic acid, hydroxysebacic acid, pentadecanoic acid, and trans-cinnamaldehyde (Fig. 3B-3C). Interestingly, we also found that the feces of patients who had FMT had a lot more dihydrosphingosine, a metabolite that is linked to nerve regeneration (Fig. 3B-3C).

Serum metabolite analysis indicated that FMT significantly influenced the patients' fatty acid metabolism, primarily by elevating levels of 3-hydroxytetradecanedioic acid and stearidonic acid, while reducing fatty acid-associated metabolites like undecylenic acid, transvaccenic acid, oleic acid, 4-hydroxyphenylacetic acid, and γ-dodecalactone (Figs. 3D-3E and S4).

### Integrated Multi-Omics Analysis Links Metabolite Shifts to Specific Microbial Texa after FMT

**Fig. 4:**
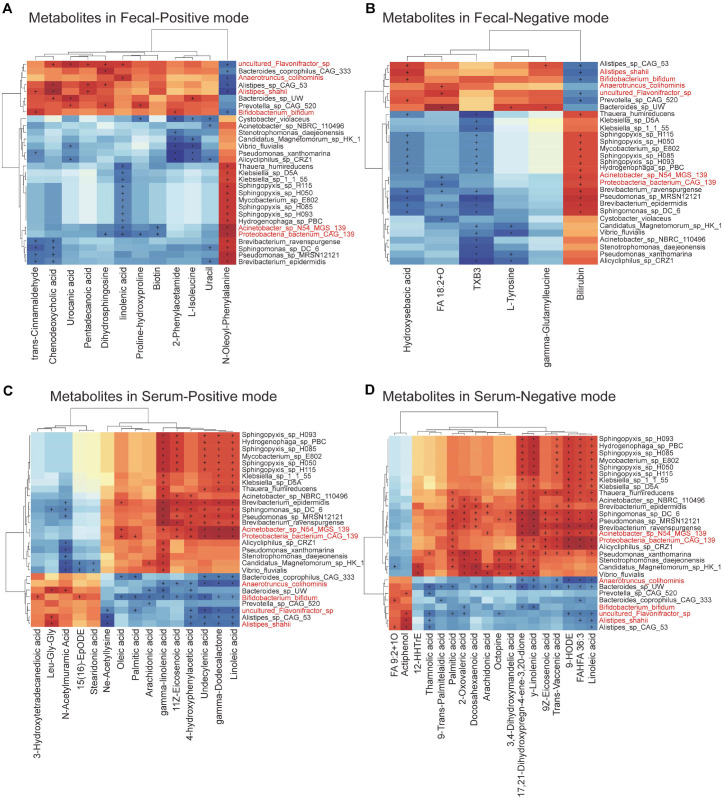
Metagenomics and metabolomics integrated analysis in the feces and serum of patients after FMT. (**A-B**) Integrated metabolomics and metagenomics were used to analyze the correlation between different bacterial strains and different metabolites in feces. (**C-D**) Integrated metabolomics and metagenomics were used to analyze the correlation between different bacterial strains and different metabolites in serum. Blue represents a negative correlation, red represents a positive correlation, and the deeper the color, the stronger the correlation. +: *P* < 0.05, *: *P* < 0.01.

We conducted a comprehensive study to ascertain the correlation between alterations in gut microbiota and metabolites and their capacity to mitigate the symptoms of PD. First, a panel of bacterial strains with a higher relative abundance was associated with lower levels of fecal N-Oleoyl-Phenylalanine and bilirubin (Fig. 4A-4B). So, lowering these two metabolites might help with the symptoms of Parkinson's disease. The decline of *Sphingomonas* sp. DC-6, *Brevibacterium epidermidis*, *Brevibacterium ravenspurgense*, and *Pseudomonas* sp. MRSN 12121, alongside the increase of *Alistopes shahii*, may be attributed to heightened concentrations of trans-cinnamaldehyde and chenodeoxycholic acid. *Bifidobacterium bifidum* may help make 2-phenylacetamide and trans-cinnamaldehyde. The reduced prevalence of *Thauera humireducens*, *Klebsiella* sp. D5A, and other strains was associated with the increased TXB3 level. The increase in L-tyrosine (Fig. 4A-4B) may have been caused by a higher number of *Bacteroides* sp. and a lower number of *Candidatus magnetmorum* sp. HK-1, *Vibrio fluvialis*, and other strains.

After FMT, we looked at the link between serum metabolite profiles and microbiota and found a strong link with changes in the levels of undecylenic acid, linoleic acid, γ-linoleic acid, oleic acid, 11Z-eicosenoic acid, γ-ldodecalactone, transvolatile acid, 4-hydroxyphenylacetic acid, and FAHFA 36:3 (Fig. 4C-4D). The reduction in oleic acid levels may be attributed to decline in *Proteobacteria bacterium* CAG 139 and *Acinetobacter* sp. N54 MGS 139, together with an increase in *B. bifidum*. Changes in other strains, on the other hand, were linked to the lower level of gamma-dodecalactone. These strains exhibited diminished abundance in *Proteobacteria bacterium* CAG 139 and *Acinetobacter* sp. N54 MGS 139, among others, and heightened abundance in *B. bifidum* and *Alistipes shahii*. The serum level of trans-volatile acid may decrease if the abundance of *Anaerotruncus colihominis* rises while that of *P. bacterium* CAG 139 and *Acinetobacter* sp. N54 MGS 139 diminishes (Fig. 4C-4D).

## Discussion

This study investigated the efficacy and undesired effects of FMT treatment in five PD patients suffering from constipation. A primary concern of this study is the safety of FMT. In one case, fecal bacteria transplantation led to severe bacteremia in two patients with dysfunctional immune systems, resulting in the death of one patient [22] Pathogenic *E. coli* that make the enzyme pathogenic extended-spectrum beta-lactamase (ESBL) were said to be the cause of bacteremia. To make sure that FMT is safe and to avoid tragedies like this, donors and recipients would need to be carefully screened. So, we looked into how patients and donors were recruited before the procedure, and we kept an eye on each one. There were no major side effects or problems during therapy, and the 8-week follow-up showed no problems either. Our initial trial comprised only five patients, yet FMT may be beneficial in treating PD, particularly motor symptoms and constipation.

We also examined changes in the constitution of commensal microbiota and metabolic alterations subsequent to FMT to assess the linkage of intestinal flora with FMT treatment for PD. Because of small sample size, we could not recognize a substantial effect of FMT on species diversity within the microbiota; however, we observed considerable variations and enduring changes in specific strains at the strain level. After transplantation, we noted a substantial increase in the prevalence of strains such as uncultured *Flavonifractor* sp., *Anaerotruncus colihominis*, *B. bifidum*, *Acinectobacter* sp., and *P. bacterium*, whereas *Acinetobacter* sp. and other strains exhibited a decrease in prevalence. By further examining the potentially microbiota-derived metabolites, we found a group of metabolites that may be connected to the pathophysiology and advancement of PD. Among these, synthesis of IAA, dopamine, and tyrosine can alleviate neurological symptoms in PD patients [23, 24]. Thromboxane (TX) B3 has been demonstrated to exhibit anti-inflammatory and anti-tumor characteristics [25]. Pentadecanoic acid has been shown to inhibit neuroinflammatory processes and is linked to neuro-immunity [26]. Maslinic acid potentially alleviates neurological symptoms and inhibit tumor proliferation by inhibiting IκB-α phosphorylation and NF-κB p65 DNA binding [27]. Animal studies and further clinical research will be undertaken to determine the direct effects of relevant strains and their metabolic products on alleviating PD symptoms. Although FMT ameliorated PD, we observed that this protective effect gradually waned with extended observation, especially in the presence of motor symptoms. There was a strong connection between the alterations in the intestinal flora and its metabolic products when the intestinal flora and its metabolites were observed at before FMT and again at the eighth week. Two and four weeks after FMT, the number of *Acinectobacter* sp., *B. bifidum*, and *Anaerotruncus colihominis* went up quickly, but then slowly went down (Fig. 2F). For chronic digestive disorders such as constipation and inflammatory bowel disease, FMT treatment often requires repetition to achieve and maintain a therapeutic effect [28, 29]. Thus, additional investigation is needed to confirm the consistency of FMT therapy for PD and intensify the efficacy of FMT treatment by selecting the optimal beneficiaries.

The constraint of this study lies in the small sample size and lack of a randomized controlled group. These limitations must be interpreted with caution. This pilot trial demonstrates that fecal microbiota transplantation is safe and may alleviate constipation and motor traits in patients with PD, likely through remodeling of the gut microbiota and modulation of fatty acid and amino acid metabolism. Although effects diminished over time, the findings highlight the therapeutic potential of FMT in PD and support the need for larger randomized controlled trials to confirm efficacy and optimize treatment strategies. In the future, a large sample randomized controlled study will be needed to clarify the effectiveness of FMT in PD treatment.

## Supplemental Materials

Supplementary data for this paper are available on-line only at http://jmb.or.kr.



## Figures and Tables

**Table 1:** Characteristics of patients included in FMT.