# Supplementary material for: Pilot Clinical Trial of Fecal Microbiota Transplantation for Constipation in Parkinson's Disease
Source: J Microbiol Biotechnol. 2025 Dec 29;35:e2509029. doi: 10.4014/jmb.2509.09029 (PMC12790986; doi:10.4014/jmb.2509.09029)
Supplement: Supplementary file 1 [file jmb-35-e2509029-supple.pdf]

J. Microbiol. Biotechnol. 2025. 35: e2509029
https://doi.org/10.4014/jmb.2509.09029

Supplementary Figures

Figure S1

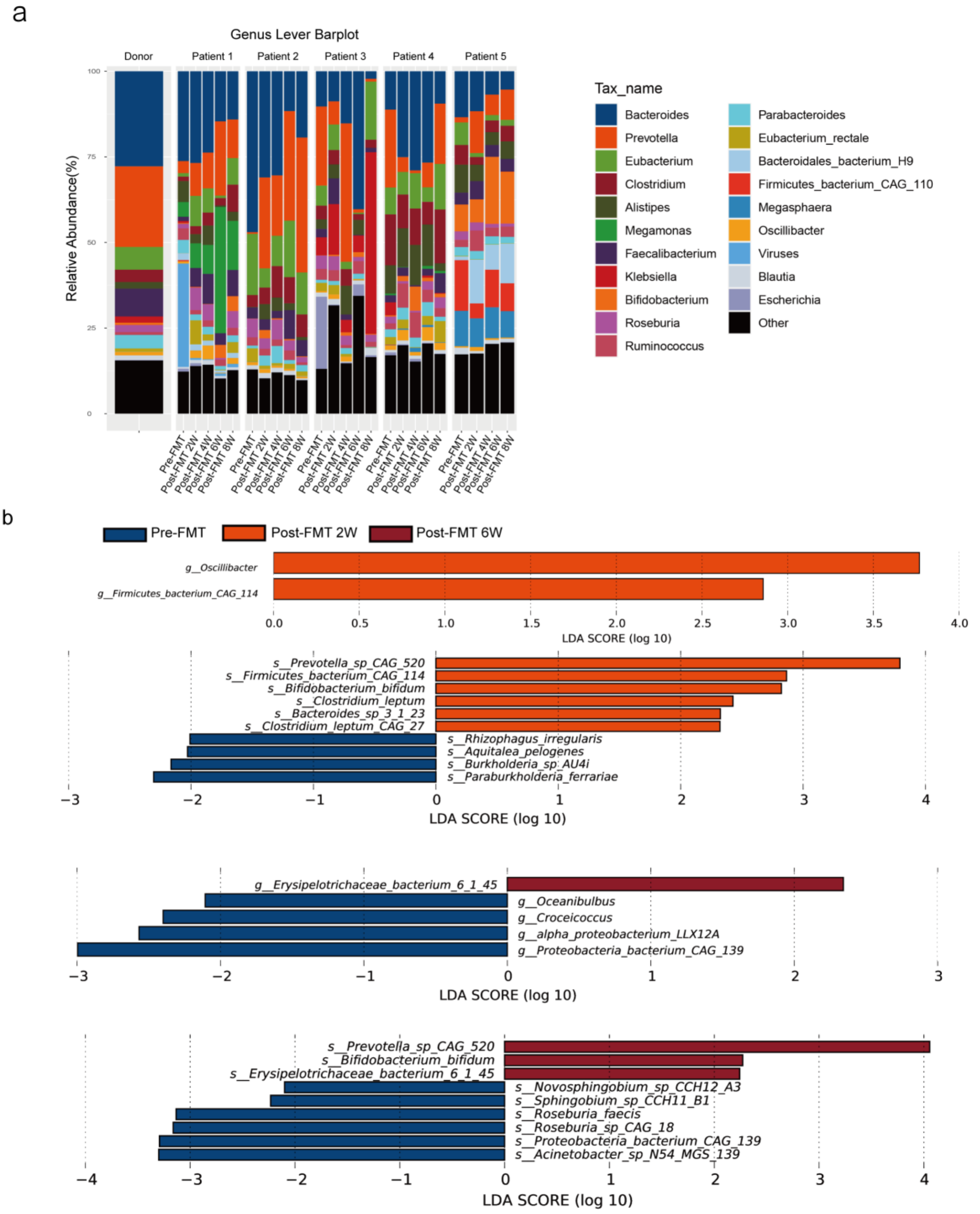

Fig. S1. FMT significantly improved constipation and neurological symptoms in Parkinson's patients.

(a) Changes in bacterial genus abundance at different time points before and after fecal bacteria transplantation;

(b) The changes in genera and strains at the 2nd and 6th weeks after and before FMT.

Figure S2

a

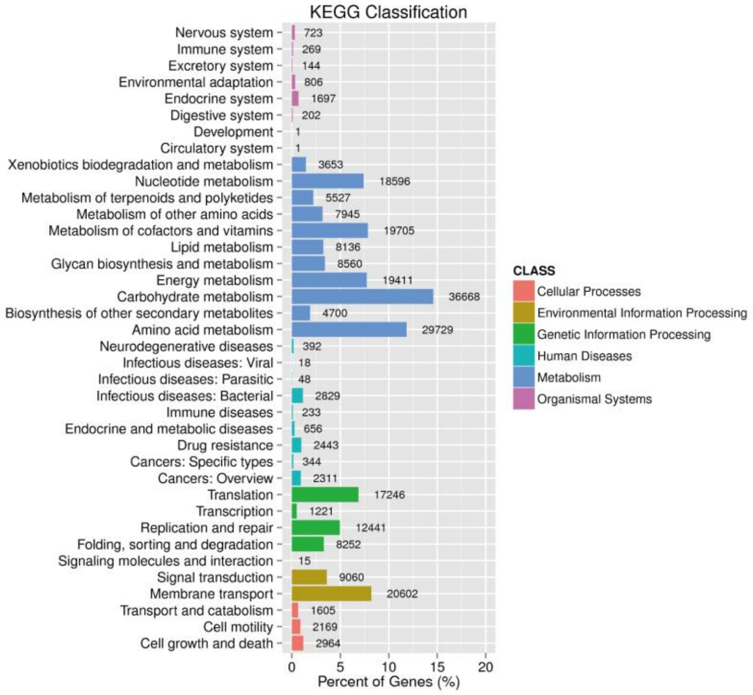

b

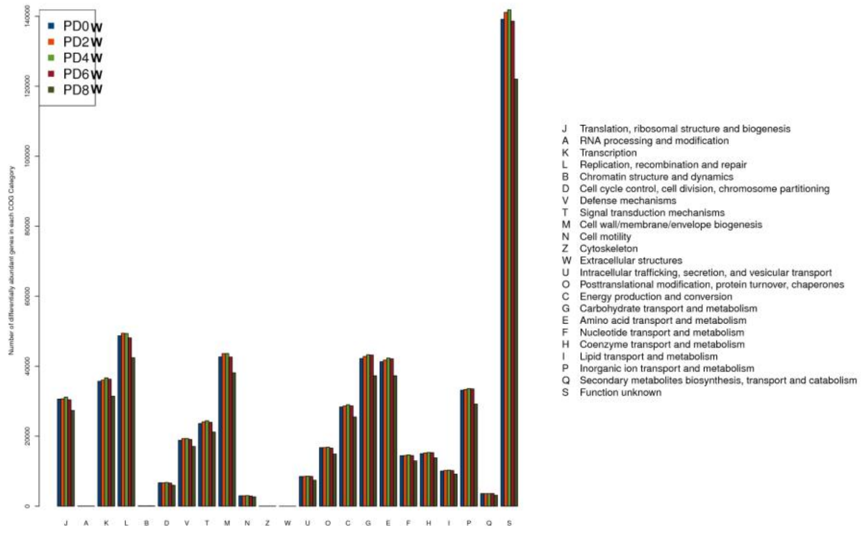

Fig. S2. Functional genomics found that fecal bacteria transplantation mainly affected the metabolic pathway of flora. (a-b) Functional genomics analysis of gene structure changes in flora after FMT. Using BLAST (Version 2.2.28+), the nonredundant genes were annotated against the KEGG (Kyoto Encyclopedia of Genes and Genomes)/EggNOG (evolutionary genealogy of genes) database. When the assembled protein sequence was similar (score≥60 and E value < 1e–5) to a protein sequence in the database, the assembled protein was considered to play the same role as the protein in the database.

Figure S3

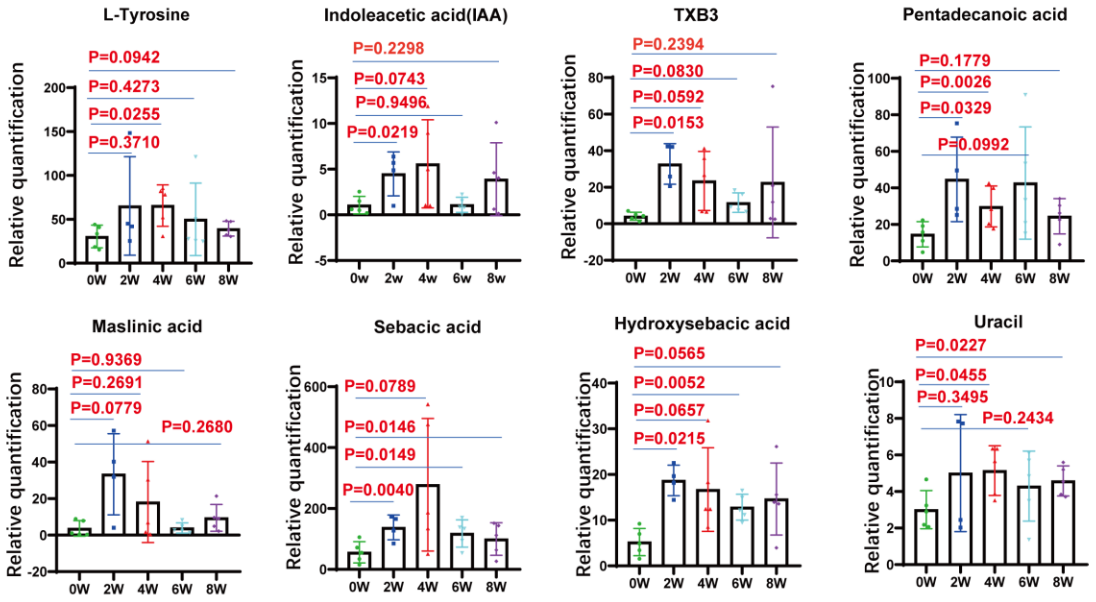

Fig. S3. Metabonomic analysis showed that the changes in differential metabolites decreased with the extension of time in feces. Metabolite analysis was performed in Prism v8.2.1 (GraphPad Software, Inc.) Paired two-tailed Student's t test was used to determine differences between groups. *P* values of <0.05 were considered statistically significant.

Figure S4

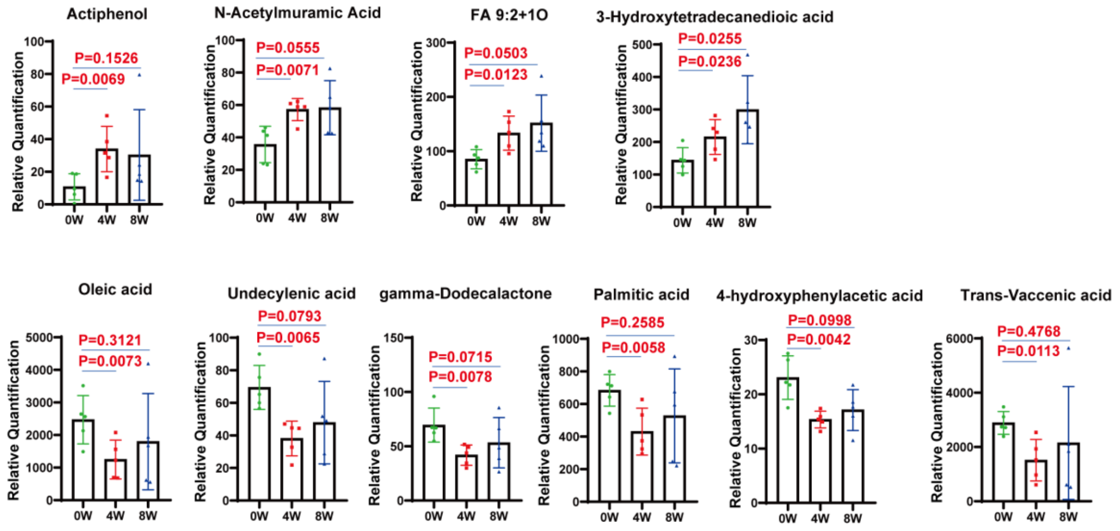

Fig. S4. Metabonomic analysis showed that the changes in differential metabolites decreased with the extension of time in serum. Metabolite analysis was performed in Prism v8.2.1 (GraphPad Software, Inc.) Paired two-tailed Student's t test was used to determine differences between groups. *P* values of <0.05 were considered statistically significant.
